# Supplementary material for: Emergent helical edge states in a hybridized three-dimensional topological insulator
Source: Nat Commun. 2022 Oct 27;13:6386. doi: 10.1038/s41467-022-33643-9 (PMC9613897; doi:10.1038/s41467-022-33643-9)
Supplement: Supplementary file 1 — Supplementary Information [file 41467_2022_33643_MOESM1_ESM.pdf]

Supplementary Information

Emergent Helical Edge States in a Hybridized Three-Dimensional Topological Insulator

Su Kong Chong[1]*, Lizhe Liu[2], Kenji Watanabe[3], Takashi Taniguchi[3], Taylor D. Sparks[2], Feng Liu[2], and Vikram V. Deshpande[1]*

[1]Department of Physics and Astronomy, University of Utah, Salt Lake City, Utah 84112 USA

[2]Department of Materials Science and Engineering, University of Utah, Salt Lake City, Utah 84112 USA

[3]National Institute for Material Science, Tsukuba, Japan

*Corresponding author: sukong.chong@utah.edu, vdesh@physics.utah.edu

---

Table of Contents

I. Gap calculations from density functional theory

II. Layer-dependent hybridization gap

III. Helical Edge States

IV. Magnetic field response on the zeroth Landau levels

V. Parallel magnetic field on hybridization gap

VI. Electric field modulation of the hybridization gap

VII. Potential topological materials for topological transistors

I. Gap Calculations from Density Functional Theory

To investigate the 2D crossover for BSTS 3D TI, we first perform DFT calculations for our BSTS compound. As shown in Supplementary Fig. 1a, we recover the 3D bulk gap of ~0.3 eV [1,2] for the BSTS. The layer-dependent surface states spectra are presented in descending order of thickness from 10 to 1 QL in Supplementary Fig. 1b-k. The extracted hybridization gaps from the surface bands show an exponential increase of the surface gap size with decreasing number of layers. We also compare the hybridization gap for the BSTS compound at different stoichiometry in Supplementary Fig. 2. Fittings of the thickness-dependent hybridization gap to the exponential relation $\propto e^{-\lambda d}$ yield a characteristic length, λ, of ~0.83 and ~0.56 nm^{-1} for $BiSbTe_{1.5}Se_{1.5}$ and $Bi_{0.7}Sb_{1.3}Te_{1.05}Se_{1.95}$, respectively. Our selected $Bi_{0.7}Sb_{1.3}Te_{1.05}Se_{1.95}$ compound shows a larger surface hybridization gap and termination at greater thickness owing to its high intrinsic bulk band compared to other compositions.

Gap parity is an important measure of topological phases. To confirm the topological nature, we followed the proposed analytical method to determine the parity of the hybridization gap. The opposite parity of +1 and -1 denote the normal and inverted gap states, respectively. We first verify the method by checking the parity eigenvalues of the well-known binary TI compounds, including Bi_2Se_3 [3], Sb_2Te_3 [4], and Sb_2Se_3 [5], as summarized in Supplementary Table 1. Similar to the previously reported works on binary TIs, the hybridization gap parity of BSTS reveal an oscillatory behavior in layer thickness where two inverted regions with odd parity were identified.

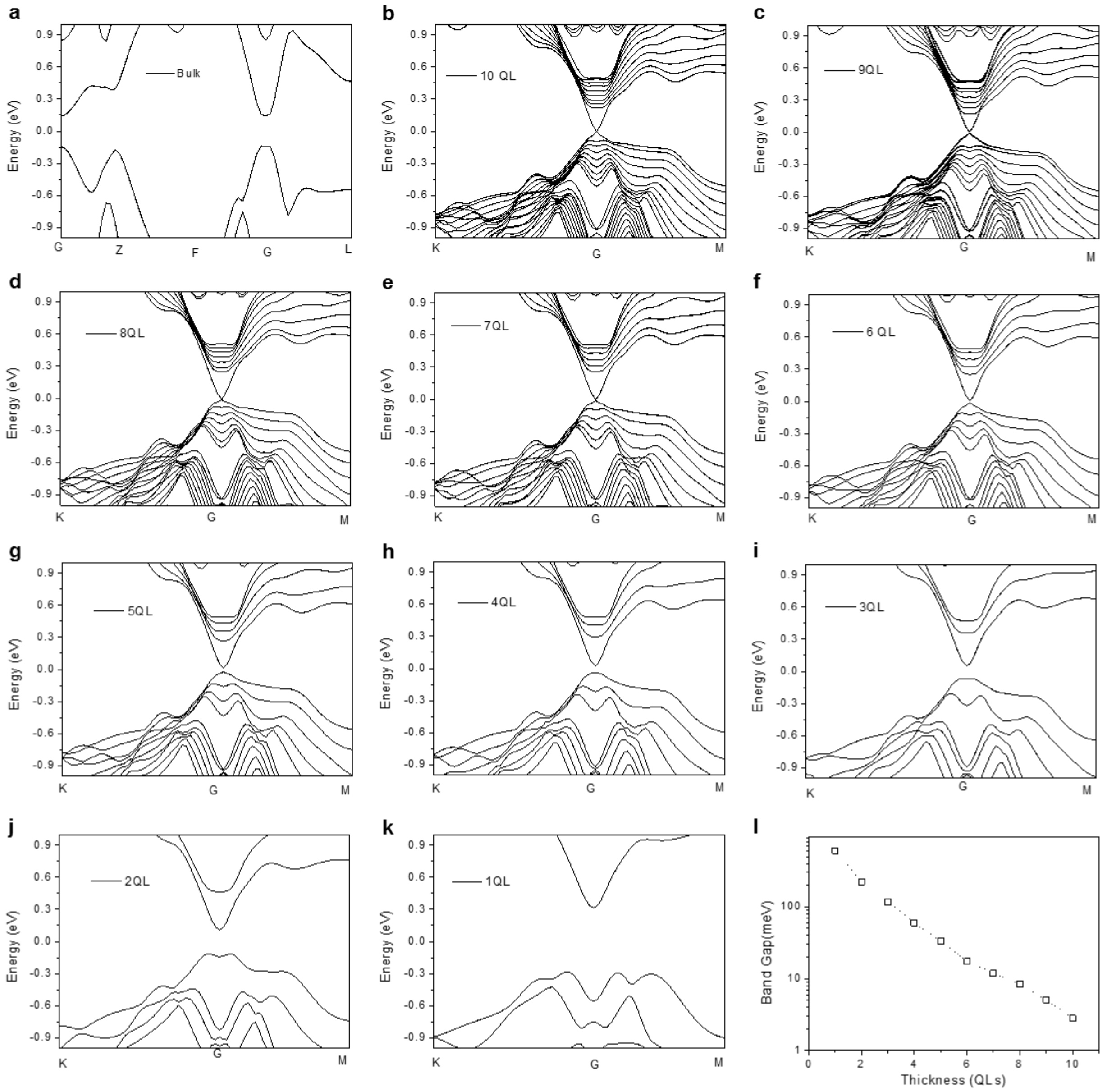

Supplementary Figure 1. DFT calculations on hybridization gap open at different layer thickness for the $Bi_{0.7}Sb_{1.3}Te_{1.05}Se_{1.95}$ (BSTS). **a** bulk band, and surface band at **b** 10 QL down to **k** 1 QL of the BSTS compound. **l** Thickness dependence hybridization gap extracted from DFT analyses.

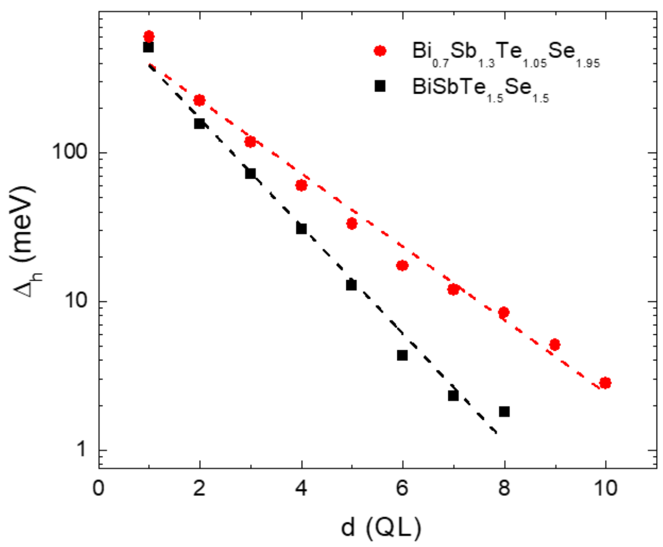

Supplementary Figure 2. Variation of hybridization gap (Δ_h) as a function of layer thickness (d) for two different compositions of hBSTS. Dashed lines in the figure are fittings to the Δ_h and d relation.

Supplementary Table 1. Gap parity calculation for different 3D TI compounds. The layer dependence parity for Bi_2Se_3, Sb_2Te_3 and Sb_2Se_3 compounds included in the table are reproduced based on the references [3-5].

Layers	1	2	3	4	5	6	7	8	9	10
Bi_2Se_3	1	1	-1	-1	-1	1	1	1	-1	-1
Sb_2Te_3	1	1	1	-1	-1	-1	---	---	---	---
Sb_2Se_3	-1	1	-1	1	1	1	1	-1	1	-1
$Bi_{0.7}Sb_{1.3}Te_{1.05}Se_{1.95}$	1	1	1	1	-1	-1	1	1	-1	1

II. Layer-Dependent Hybridization Gap

Supplementary Table 2 lists the variable thickness hBSTS devices fabricated into a Hall bar geometry for the studies. These devices were fabricated in a dual-gated configuration with top and bottom gates made of hBN and Gr flakes. For monolayer hBSTS, the flakes were too small for the Hall bar structure, therefore were fabricated in a two-terminal contact.

Thermal Activation Energy

Supplementary Fig. 4 shows the temperature and gate-dependent R_{xx} for the variable thickness hBSTS. The activation behavior at their CNPs is due to the intersurface hybridization gap. While the R_{xx} is greatly suppressed (G greatly enhanced) at high charge density as the chemical potentials are tuned into the electron and hole conduction regimes. This behavior is very similar to a semiconductor, suggesting a trivial insulating state for these samples. Supplementary Fig. 5 shows the Arrhenius plots for different thickness hBSTS samples. The fittings for G_{xx} *versus* temperature curves yield the thermal activation energy (E_A) for the variable thickness hBSTS as summarized in Fig. 2d.

Differential Conductance

Differential conductance (dI/dV) spectra were measured in a two-terminal probe using the main source-drain contacts. Supplementary Fig. 6 shows the dI/dV *versus* V_b curves at the CNPs for different thickness BSTS. The hybridization gaps were determined from the turn-on voltage typically at conductance in the order ~e^2/h, as marked by the red arrows in the figures. We used the two-slopes method to better evaluate the turn-on voltage for gap size estimation as indicated by the blue and pink dashed lines in Supplementary Fig. 6. To exclude the nonlinear current-voltage signal from the bad contact effect, we also measured the dI/dV in the conduction region by changing the gate voltages for all samples.

Supplementary Table 2. Device specifications. List of the thickness (d), length (L), width (W), and geometry of the BSTS dual-gated devices fabricated for the studies.

d (nm)	L (±0.1 μm)	W (±0.1 μm)	Geometry
1	1.0	2.0	two terminals
3	2.8	2.3	four terminals
6	5.4	3.7	Hall bar
7	3.3	2.3	Hall bar
8	4.7	2.8	Hall bar
9	5.1	3.4	Hall bar
10 (S1)	3.0	3.2	Hall bar
10 (S2, no gap)	4.5	5.5	Hall bar
10 (S3)	1.0	1.3	Hall bar
12	6.0	5.0	Hall bar

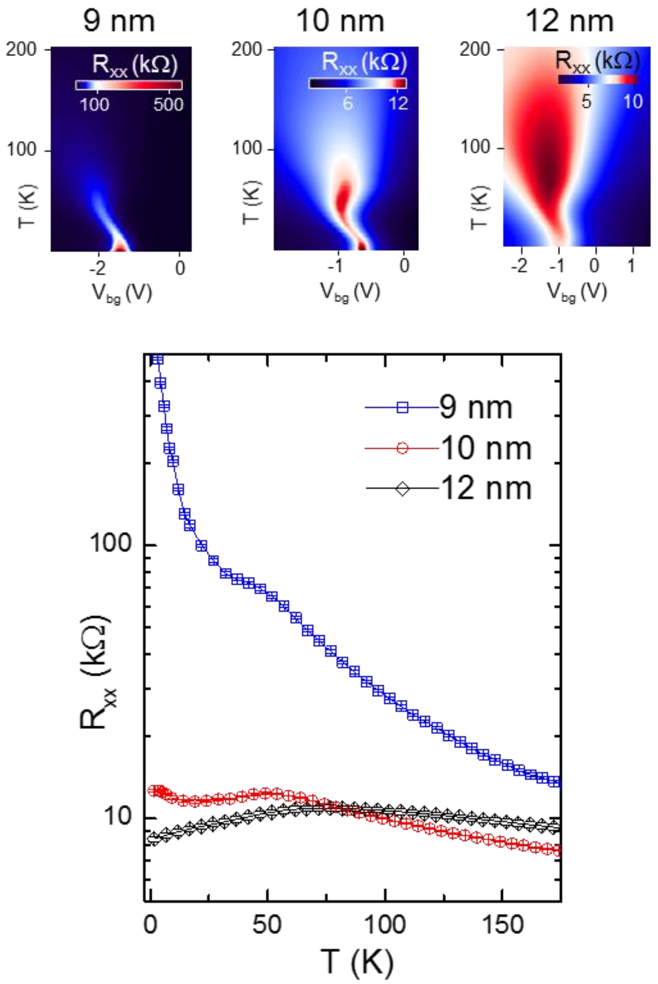

Supplementary Figure 3. Temperature-dependent transport for BSTS 3D TI with thicknesses near the 2D crossover regions. (top) Color maps of R_{xx} as functions of temperature and gate voltage for the 9 nm, 10 nm and 12 nm BSTS. (bottom) The R_{xx} versus temperature curves extracted from the color maps at the charge neutrality for the 9 nm, 10 nm and 12 nm BSTS devices. For BSTS thickness above 10 nm, the R_{xx} gradually decreases at lower temperature due to the gapless surface states.

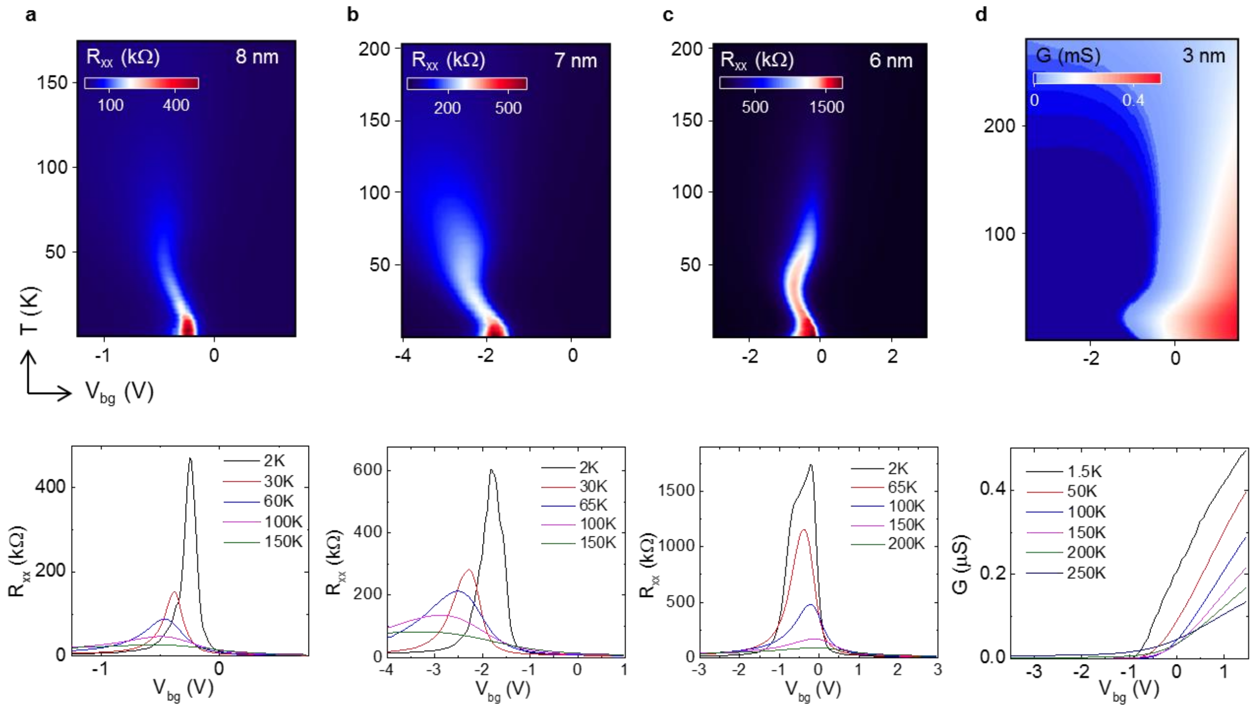

Supplementary Figure 4. Temperature and gate-dependence transport for the hBSTS. Color maps (top) and line profiles (bottom) of R_{xx} (or G) versus V_{bg} at different temperature for the **a** 8, **b** 7, **c** 6, and **d** 3 nm hBSTS. Note that the color map of the 3 nm hBSTS is plotted in two-probe conductance (G) as the four-probe resistance measurements are irrelevant due to its extremely large in-gap resistance.

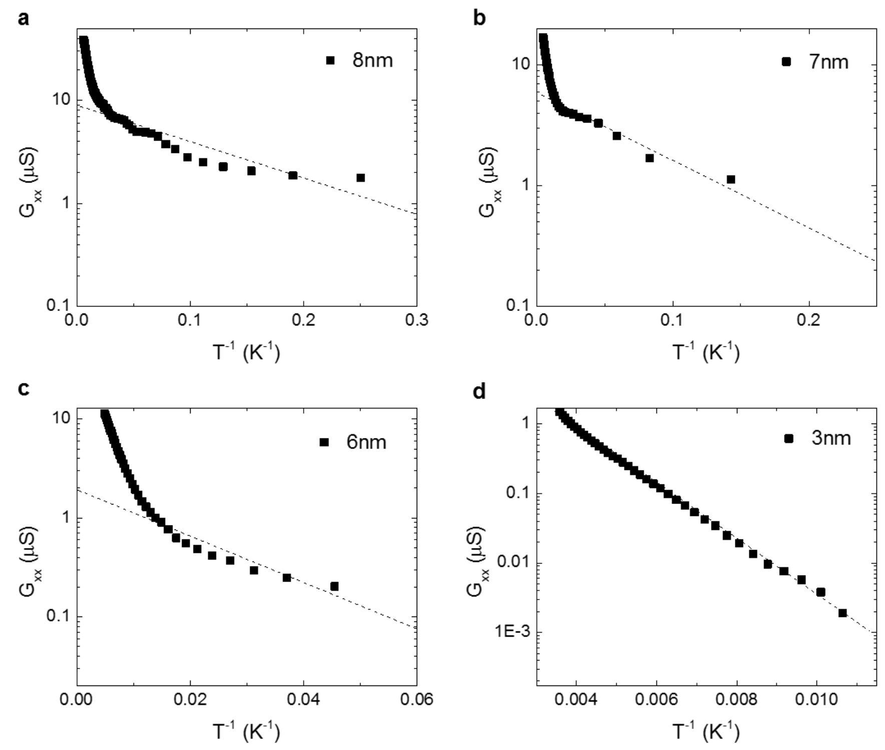

Supplementary Figure 5. Arrhenius plots. Comparison of G_{xx} versus T^{-1} curves for the hBSTS at different layer thickness of **a** 8, **b** 7, **c** 6, and **d** 3 nm. Dashed lines in the figure are fittings to extract the thermal activation energies (E_A) for the respective thickness.

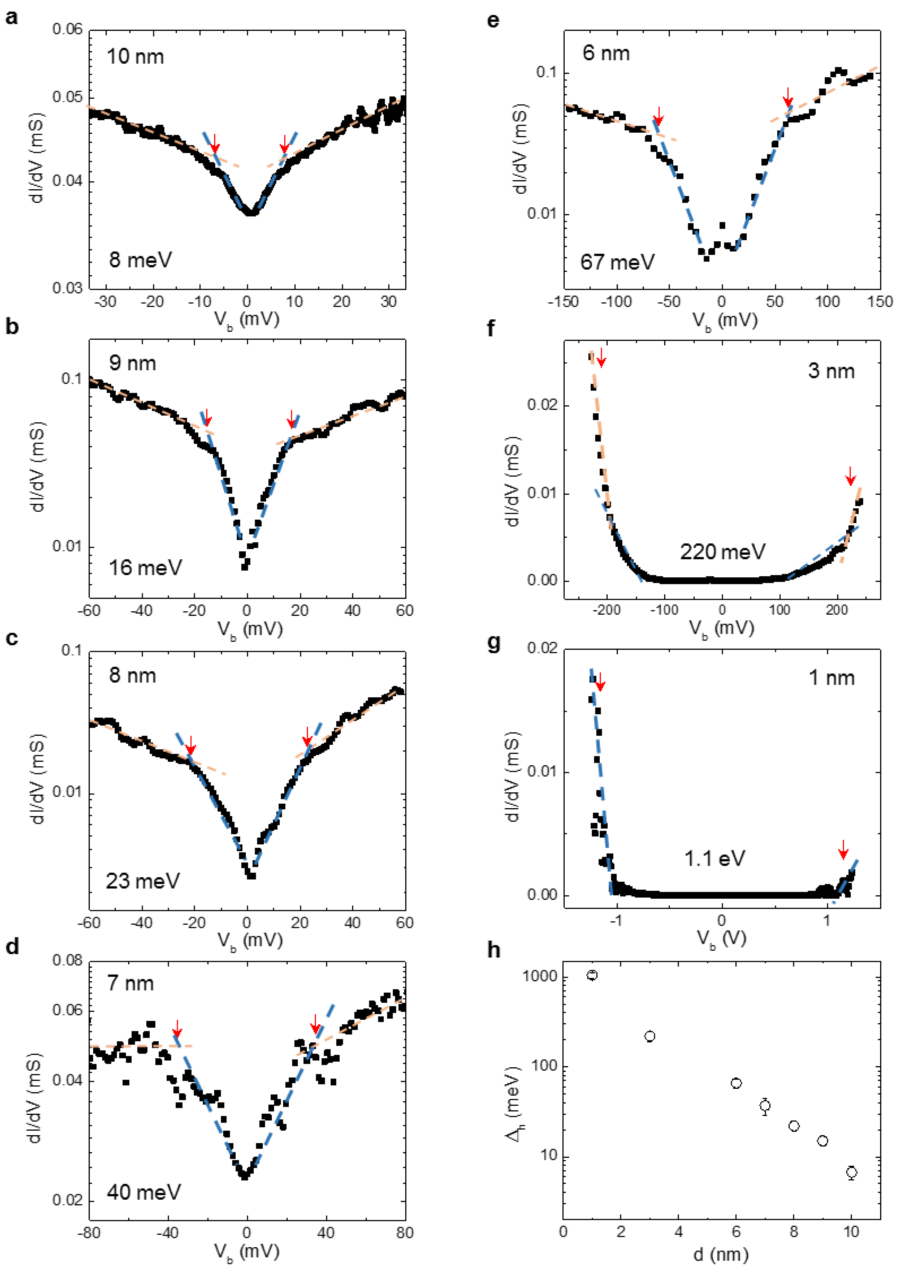

Supplementary Figure 6. Layer dependence hybridization gap evaluated by differential conductance. Plots of dI/dV *versus* V_b for the hBSTS with thicknesses of **a** 10, **b** 9, **c** 8, **d** 7, **e** 6, **f** 3, and **g** 1 nm. The red arrows in the figures point to the turn-on voltages. **h** Plot of Δ_h versus flake thickness determined from the dI/dV *versus* V_b spectra. Error bars in **h** are the standard deviation from the fittings.

III. Helical Edge States

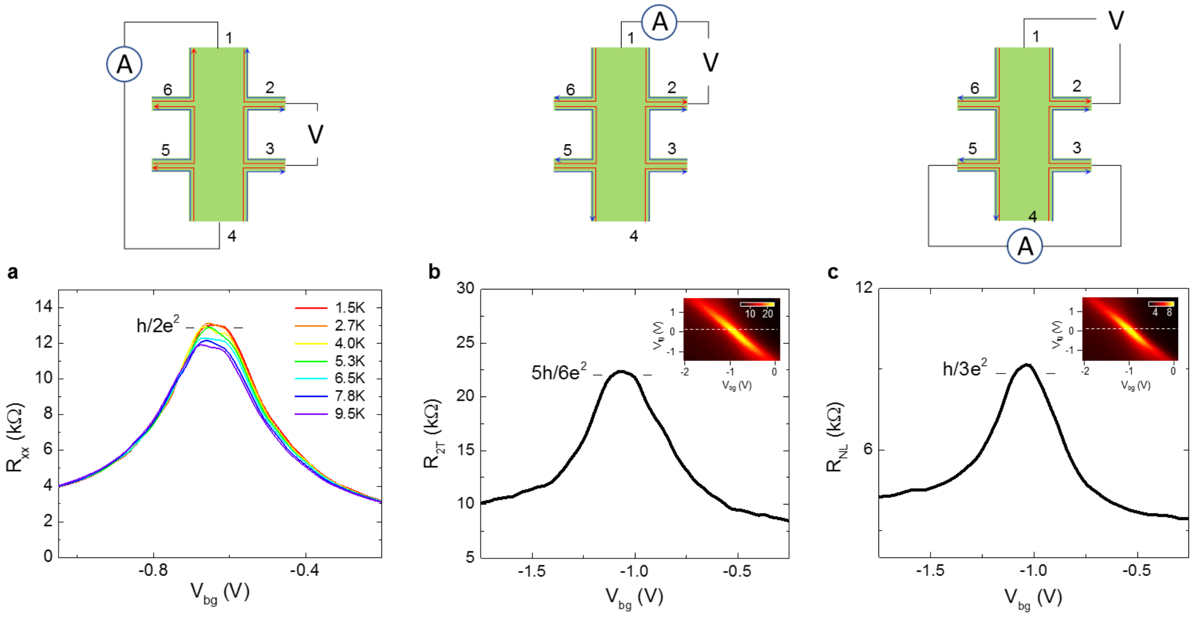

Supplementary Figure 7. Gate dependent helical edge states transport. (top) Schematics of the measurement configurations for **a** Longitudinal resistance (R_{xx}), **b** two-terminal resistance (R_{2T}), and **c** non-local resistance (R_{NL}) for the 10 nm hBSTS Hall bar (S1) device. (bottom) **a** R_{xx}, **b** R_{2T} and **c** R_{NL} as a function of V_{bg} measured **a** at different temperatures, **b** and **c** at 2K. Insets in **b** and **c** are the dualgate maps of R_{2T} and R_{NL}, respectively. The expected values for the helical edge transport calculated from Landauer-Buttiker formalism [6] are $R_{xx} = h/2e^2$, $R_{2T} = 5h/6e^2$, and $R_{NL} = h/3e^2$.

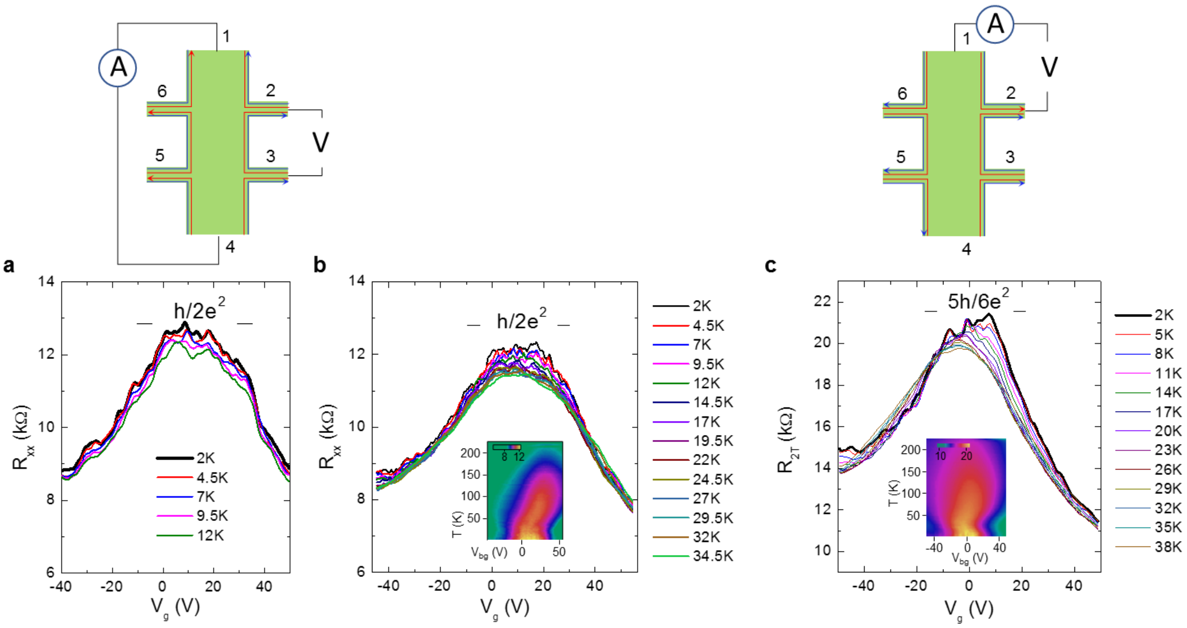

Supplementary Figure 8. Transport in an additional 10 nm hBSTS (S3) device. (top) Schematics of the measurement configurations for **a**, **b** Longitudinal resistance (R_{xx}) and **c** two-terminal resistance (R_{2T}). (bottom) **a, b** R_{xx} measured in the first and second cooldown cycles, respectively, and **c** R_{2T} measured in the second cooldown as a function of V_{bg} at different temperatures. The deviation in the R_{xx} maximum in the second cooldown cycle is attributed to a shift in the chemical potential of the top surface upon exposure to ambient. However, the weak and saturating temperature-dependent resistance was still observed. The weak temperature dependence of the in-gap R_{xx} indicates that the sample is in a 2D topological phase. Insets in **b** and **c** are the color maps of R_{xx} and R_{2T}, respectively, as function of temperature and V_{bg}. The maxima of R_{xx} and R_{2T} approach the expected values for the helical edge transport of $R_{xx} = h/2e^2$ and $R_{2T} = 5h/6e^2$, which further indicates the emergence of 2D topological states in the hBSTS.

Supplementary Table 3. Summary of the resistances measured at different configurations for the 10 nm hBSTS devices. The error bar of the in-gap resistance (R_{exp}) is the standard deviation calculated for 2-5 gate sweeps. The R_{theory} are the values calculated from Landauer-Buttiker formalism.

**Devices**	**Configurations**	**R_{exp} (kΩ)**	**R_{theory} (kΩ)**
10 (S1)	$R_{14,23}$	12.8±0.4	12.906
	$R_{12,12}$	21.9±0.4	21.150
	$R_{13,13}$	35±0.5	34.416
	$R_{35,12}$	8.8±0.3	8.604
10 (S3)	$R_{14,23}$	12.2±0.1	12.906
	$R_{12,12}$	20.8±0.3	21.150

IV. Magnetic Field Response on the Zeroth Landau Levels

A consequence of intersurface hybridization on LLs spectrum is the splitting of zeroth LLs in a perpendicular magnetic field [7,8]. As the zeroth LLs are pinned to the edges of the highest valence and lowest conduction bands, the zeroth LLs' energy E_0 can be treated as the hybridization gap Δ_h [9]. Supplementary Fig. 9a and c show the σ_{xx} versus V_{bg} plots for the 9 and 8 nm hBSTS, respectively. The two N= 0 LL peaks developed at high magnetic field are traced by dashed lines with the change in magnetic field. The developed zeroth quantum Hall plateaus together with the normal integer plateaus at high magnetic field (Supplementary Fig. 9b and d) further confirms the analyses.

To determine the E_0 change in magnetic field, we measured the quantum capacitance using a capacitance bridge method [10]. Supplementary Fig. 10a shows the mapping of total capacitance (C) as functions of V_{bg} and magnetic field for the 9 nm hBSTS. The C dip at the CNP is corresponding to the lowest density of state resulting from the hybridization gap. The dip size develops with magnetic field. For further analysis on the C dip, we extracted the chemical potential (μ) versus density (n) relation (Supplementary Fig. 10b) by integrating the quantum capacitance over the charge density. The step height in μ determines the E_0. Supplementary Fig. 10c plots the E_0 as a function of magnetic field for the 9 and 8 nm hBSTS. The E_0 is plotted at high magnetic field (> 8T) where the N= 0 LLs are fully developed. The development of E_0 in magnetic field indicates a gap widening at ~1.2 and ~1.6 meV/T for the 9 and 8 nm hBSTS, respectively.

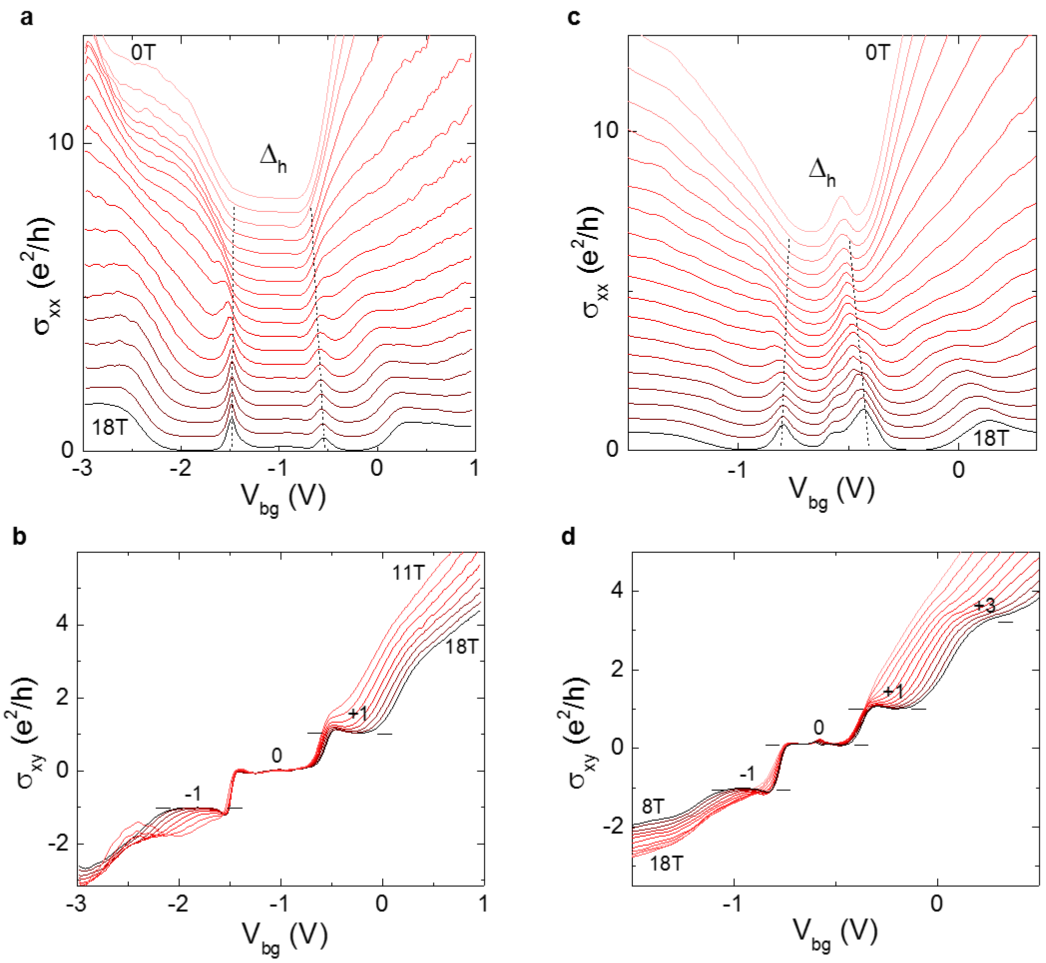

Supplementary Figure 9. Magneto-transport. Line profiles of **a**, **c** σ_{xx} and **b**, **d** σ_{xy} as a function of V_{bg} at different magnetic field for the 9 nm and 8 nm hBSTS, respectively. Dashed lines in **a** and **c** trace the developments of N= 0 sublevels with magnetic field. The filling factors of the respective LLs are labeled in **b** and **d**.

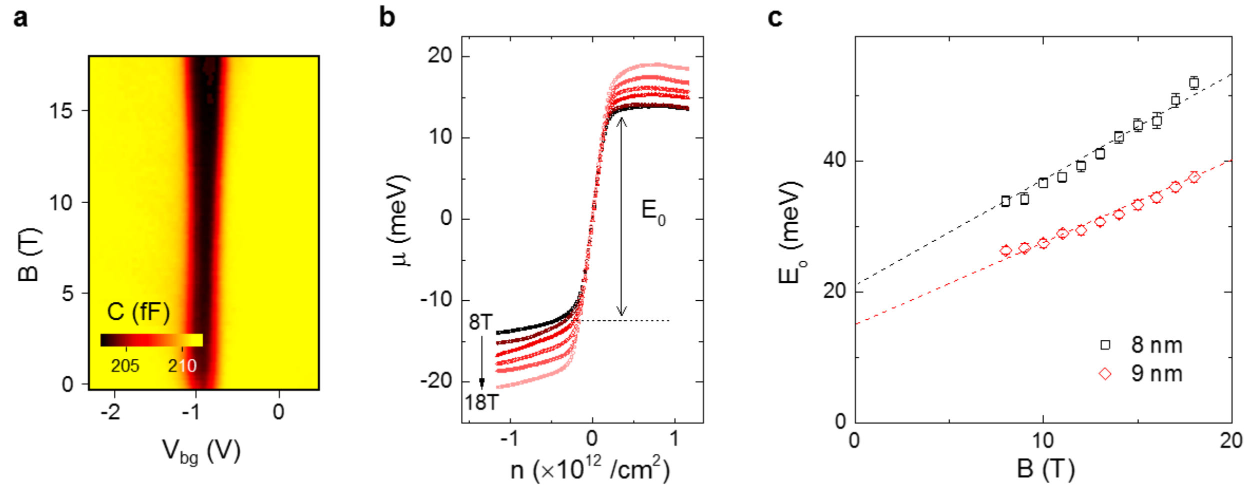

Supplementary Figure 10. Zeroth LL's energy. **a** Color map of the C as functions of V_{bg} swept across the zeroth LLs at different magnetic fields for the 9 nm hBSTS. **b** Plots of μ(n) versus n across the zeroth LL regime at different magnetic fields. The E_0 is extracted from the step height of the μ(n) at n= 0 cm^{-2}, as indicated by the arrow line. **c** Plots of E_0 as a function of magnetic field for the 8 and 9 nm hBSTS. Error bars in **c** are the standard deviation from the fittings. Dashed lines in **c** are fittings to the E_0 *versus* B relation.

V. Angular Magnetic Field on Hybridization Gap

Besides, we performed transport measurements in a parallel magnetic field. Supplementary Fig. 11 compares the R_{xx} as a function of V_{bg} at different magnetic field applied in parallel to the sample for the 9 and 10 nm hBSTS. The G_{xx} *versus* parallel magnetic field taken at their charge neutralities are inserted in the figures. Similar to the perpendicular magnetic field, the G_{xx} curve for the 9 nm hBSTS remains low and unchanged with the increase of the parallel magnetic field. Whereas for the 10 nm hBSTS, the G_{xx} curve at low field follows a similar trend as in the perpendicular magnetic field. The G_{xx} slowly decreases from ~$e^2/2h$ at a small magnetic field as a result of time-reversal symmetry breaking. The dG_{xx}/dB of ~0.01-0.02 $e^2/h/T$ (B= 0-2T) is more than one order of magnitude smaller compared to the slope in perpendicular magnetic field. While at higher magnetic field the dG_{xx}/dB changes sign and G_{xx} curve continues increasing with further increment in magnetic field. Different from the perpendicular magnetic field response which develops into QH gapped state, the parallel magnetic field can shift the surface bands' energy according to their spin states and turns into a semimetal state when the magnetic energy exceeding the hybridization gap [11,12].

This different response in parallel and perpendicular directions at high magnetic field suggests an angular magnetic field dependence transition between the two limits. To further study this transition, we plotted R_{xx} color map as functions of V_{bg} and magnetic field direction (θ) at 18 T in Supplementary Fig. 12a. The color map clearly shows suppression of QH effect as the $\theta < 20^o$. The line profile of ρ_{xx} versus θ taken at charge neutrality is plotted in Supplementary Fig. 12b. As shown in the figure, the angular magnetic field dependence R_{xx} curve starts to deviate from the oscillatory function of cos θ at $-12^o \leq \theta \leq 12^o$. At smaller θ, the R_{xx} curve reduces almost linearly

with a decrease in θ. This interesting linear dependency in angular magnetic field deserves further theoretical investigations.

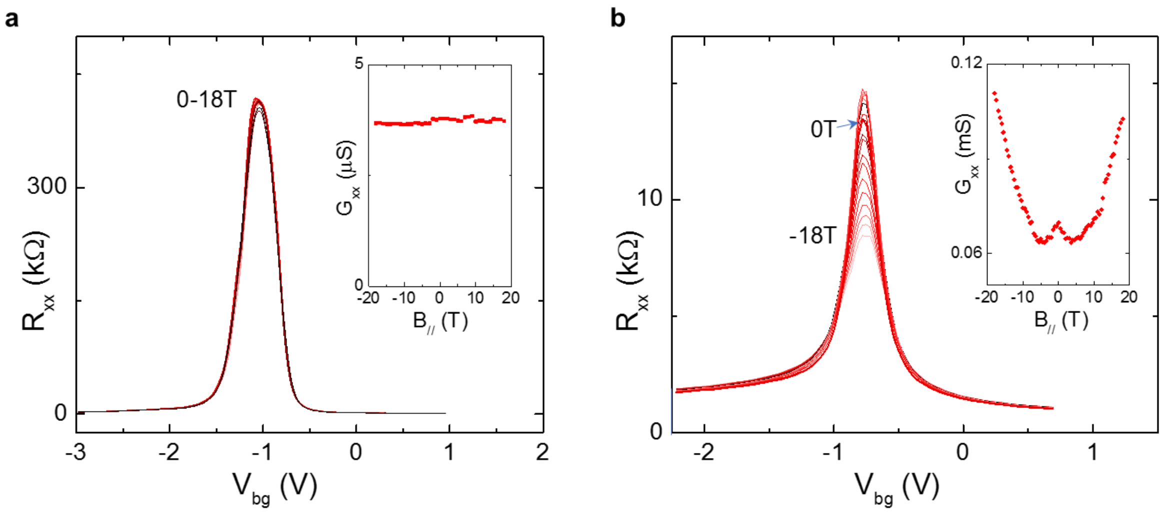

Supplementary Figure 11. In-plane magnetic field effect. Plot of R_{xx} as a function of V_{bg} measured at different in-plane magnetic fields from 0 to 18 T for the **a** 9, and from 0 to -18T for the **b** 10 nm hBSTS. Insets in **a** and **b** are plots of G_{xx} minima as a function of the in-plane magnetic field. The increasing of G_{xx} at strong in-plane magnetic field indicates a hybridization gap-closing feature as a resultant of the shifting in surface bands.

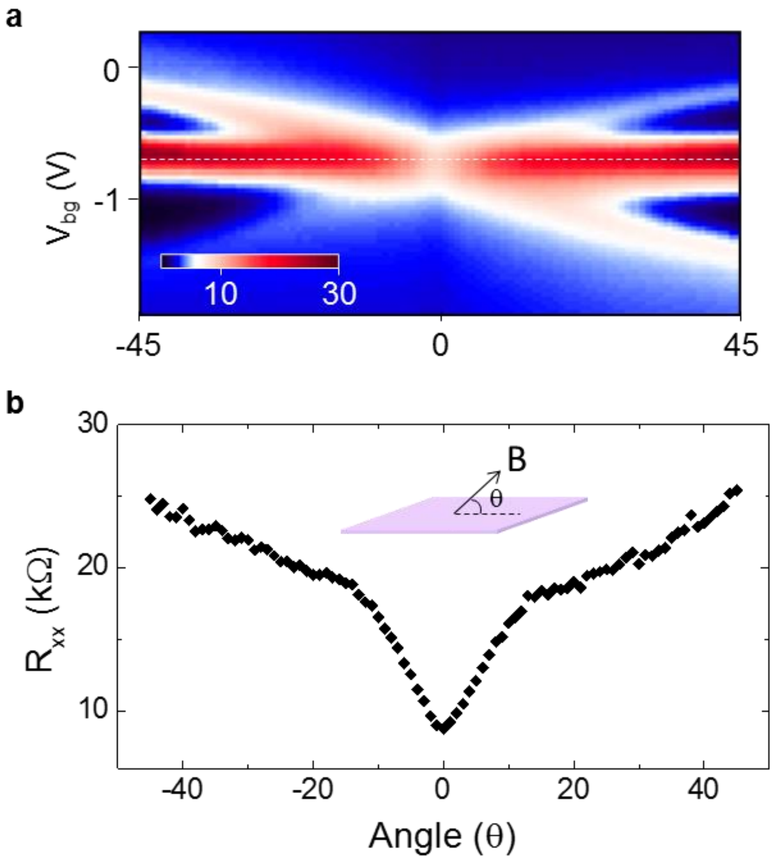

Supplementary Figure 12. Angular dependence magnetic field effect. **a** Color map of R_{xx} as functions of V_{bg} and direction of magnetic field (θ) measured at magnetic field of -18 T for the 10 nm hBSTS. **b** Line profile of R_{xx} as a function of θ taken at chemical potential aligned to the surface gap. Schematic diagram illustrates the direction of magnetic field applied to the hBSTS sample.

VI. Electric Field Modulation of the Hybridization Gap

DFT Calculations

Theoretical calculations proposed that an external electric field can induce topological phase transition by closing and reopening of the surface hybridization gap [13-15]. To investigate this effect, we first perform DFT calculations for our BSTS compound. Supplementary Fig. 13 shows the surface band structure of a 3 QL hBSTS (Δ_h~120 meV) under different external electric fields. We observe a gap-closing at a critical electric field of ~0.06 V/Å, where the Rashba-split surface bands cross at their band edges. When exceeding the critical electric field, surface band edges further separate and result in a gap-opening. We further analyze the gap parity in electric field as shown in Supplementary Fig. 14. The 3 QL hBSTS has an even parity gap, which belongs to a normal gap state. The gap parity changes sign at the crossing point and remains inverted with further increase in electric field. The gap size extracted from the calculations (Supplementary Fig. 14b) shows a slower gap-reopening rate compared to its closing rate. Furthermore, we analyze the gap parity change in electric field for 4 and 5 QL hBSTS with normal and inverted gaps, respectively, as shown in Supplementary Fig. 14a. For both cases, the gap parity flips its sign at the critical electric field, suggesting an interchange of the normal and inverted surface gap.

Dual-Gating Effect

To analyze the effect of electric field on the hybridization gap, we performed dual-gating measurement for the variable thickness BSTS samples. The results are presented in color maps of R_{xx} as functions of dual-gate voltages in Supplementary Fig. 15a-f. The diagonal feature in dual-gating maps justifies the strong coupling between the top and bottom surfaces [16]. The line profiles of R_{xx} *versus* V_{bg} taken at different V_{tg} for the respective thicknesses are shown in Supplementary Fig. 15g-l. The substantial change in R_{xx} when tuned away from charge neutrality by V_{tg} for thinner

samples indicates that they are very responsive to electric field modulation. To verify this, we convert the dual-gate voltages into displacement field (D) and further investigate the change in conduction and gap size with D.

Thermal Activation Energy

To analyze the thermal activation in electric field, we extracted the temperature-dependent ρ_{xx} at different D as plotted in Supplementary Fig. 16a, for the 9 nm hBSTS. The activation behavior slowly suppressed as D increases. At large D, ρ_{xx} curve tends to flatten at low temperature. The D-dependent hybridization gap can be evaluated from the thermal activation energy calculated at different D. Supplementary Fig. 16b shows Arrhenius plots of G_{xx} at different D, where the dashed lines are the fittings to the Arrhenius equation. The insignificant change in G_{xx} with the temperature at large D indicates again a suppression in hybridization gap by D. The activation energy E_A at different D is summarized in Fig. 4f in the main text.

Differential Conductance

The D-dependent hybridization gap can also be probed by differential conductance measurement. Supplementary Fig. 17a shows the color maps of dI/dV as a function of V_b and V_{bg} at different V_{tg} for the 9 nm hBSTS. Shifting of the dI/dV minimum towards negative polarity V_{bg} with increasing of positive V_{tg} verifies the probed hybridization gap state along the direction of the increasing D as pointed in dual-gate map in Supplementary Fig. 15a. The vanishing dI/dV minimum at high V_{tg} as indicated by the color contrast supports the thermal activation results, where the gap size diminishes at large D. Line profiles of dI/dV *versus* V_b taken at CNPs for different Vtg are plotted in Supplementary Fig. 17b. The gap size determined from the dI/dV dips at different D is included in Fig. 4f in the main text.

Quantum Capacitance

As the thermally-activated gap can be affected by the smearing effect [17], we further implement capacitance measurement to probe the thermodynamics density of states for D dependent hybridization gap. Supplementary Fig. 18 shows the color maps of total capacitance as functions of dual-gate voltages for different thickness hBSTS. The substantial reduction in C_Q dip intensity again supports the suppression of the hybridization gap at large D. The quantum capacitances C_Q at n= 0 cm^{-2} along D were extracted using the analyses discussed in our previous work [10]. The chemical potential curves were obtained by integrating C_Q^{-1} over the charge density at different D, as shown in Supplementary Fig. 19. Likewise, the hybridization gap sizes at different D are assessed quantitatively from the step heights of the $\mu(n)$ curves, as summarized in Fig. 4f in the main text.

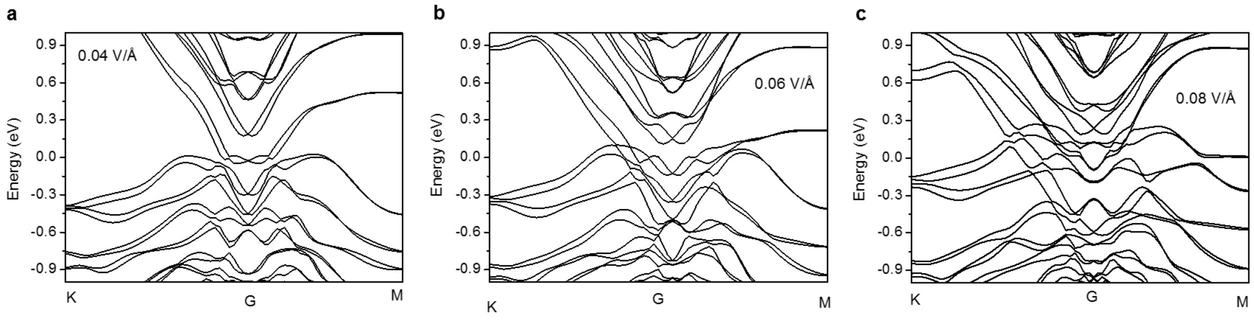

Supplementary Figure 13. Surface band evolution and hybridization gap modulation by external perpendicular electric field. Band structure of a 3 QL hBSTS simulated at electric fields of **a** 0.04 V/Å, **b** 0.06 V/Å, and **c** 0.08 V/Å.

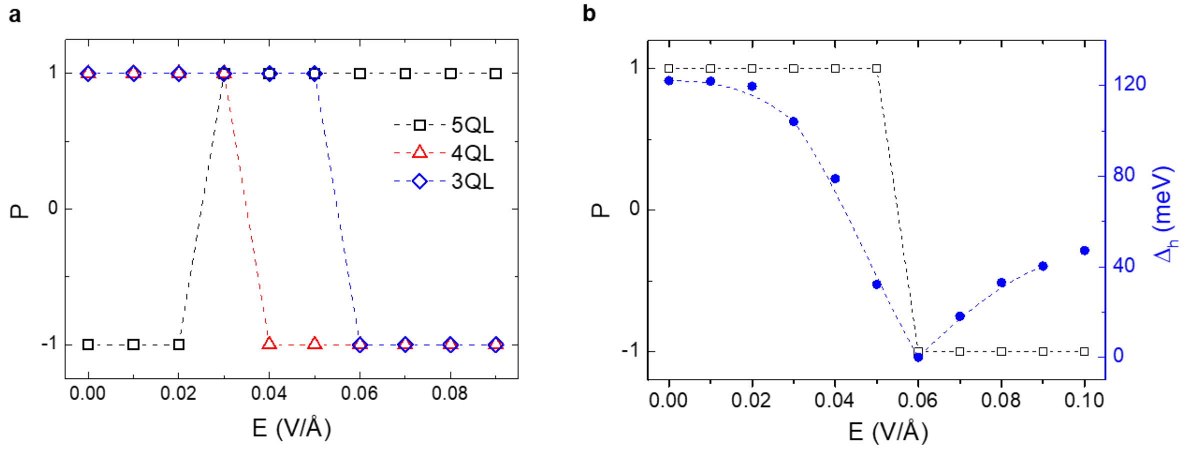

Supplementary Figure 14. Size and parity of the hybridization gap modulated by external electric field. **a** Surface gap parity as a function of electric field for different thickness hBSTS. **b** Variation of the hybridization gap size and the corresponding parity as a function of electric field for the 3 QL hBSTS.

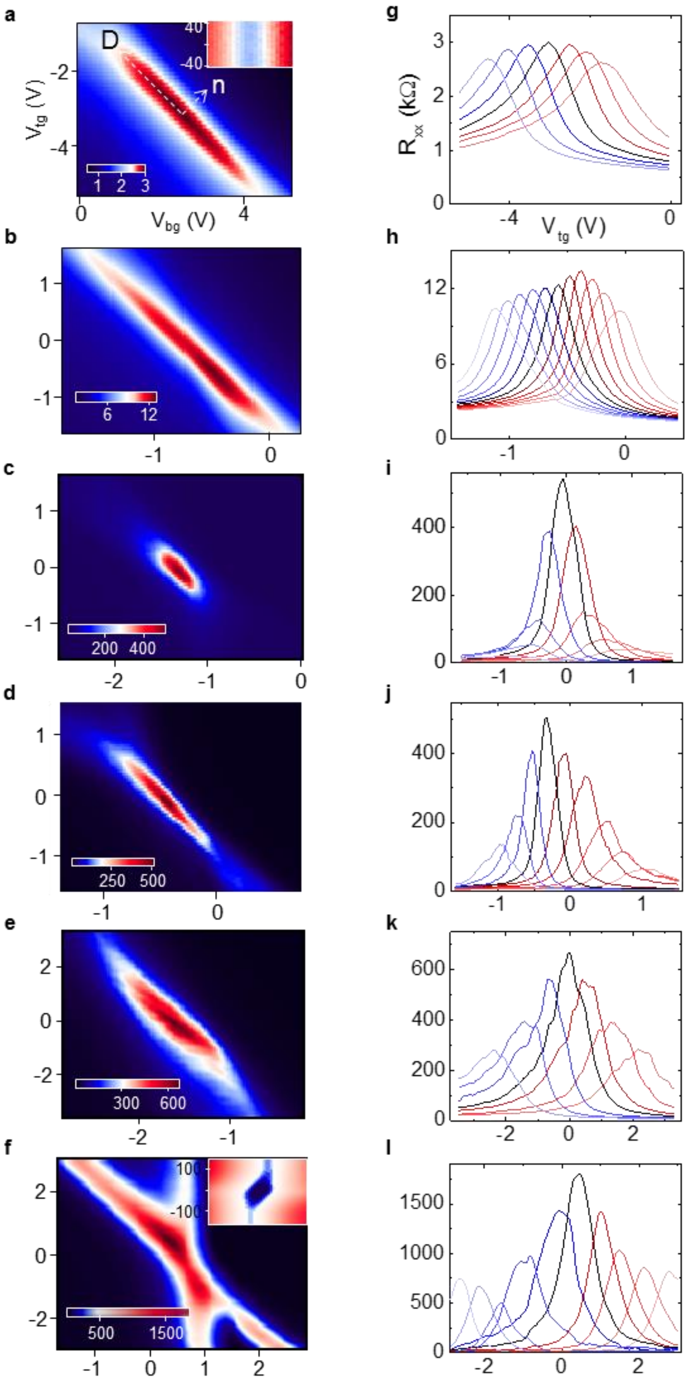

Supplementary Figure 15. Dual-gating effect. Color maps of R_{xx} as a function of dual-gate voltages at the temperature of 1.6 K for the variable thickness BSTS **a** 10 (S2, no gap), **b** 10 (S1), **c** 9, **d** 8, **e** 7, **f** 6 nm. **g-l** R_{xx} versus V_{tg} plots taken at different V_{bg} for the respective thickness samples. Insets in **a** and **f** are dI/dV maps as functions of V_b and V_{bg} for the 10 (no gap) and 6 nm BSTS, respectively.

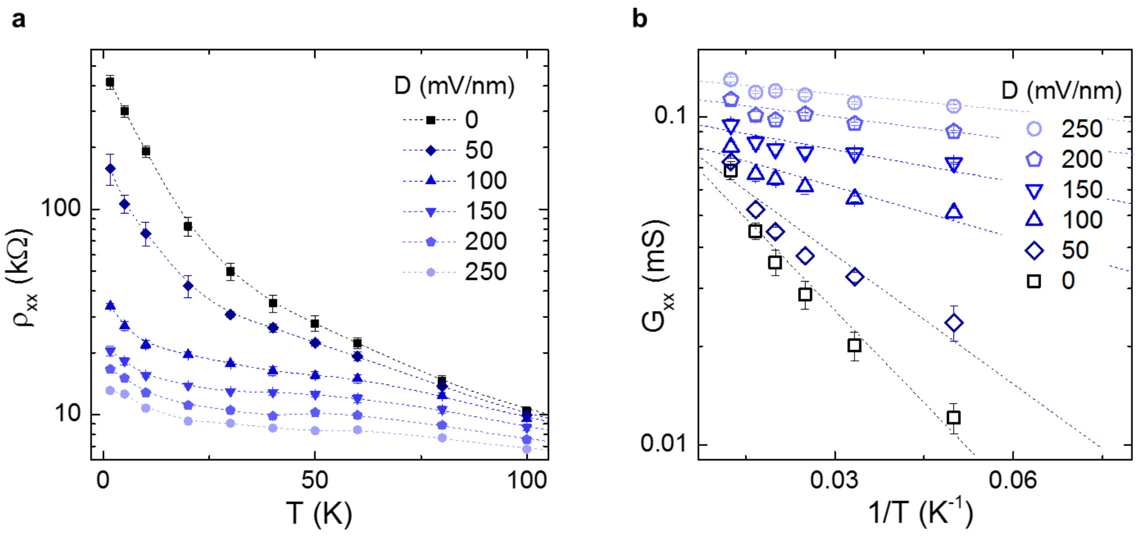

Supplementary Figure 16. Temperature dependence transport and thermal activation in displacement field. Plots of **a** ρ_{xx} *versus* T, and **b** G_{xx} *versus* T^{-1} for the 9 nm hBSTS at different D. Error bars in **a** are the standard deviation of the extracted ρ_{xx} values. Dashed lines in **b** are fittings of the G_{xx} to the Arrhenius equation. Error bars in **b** are the standard deviation from the fittings.

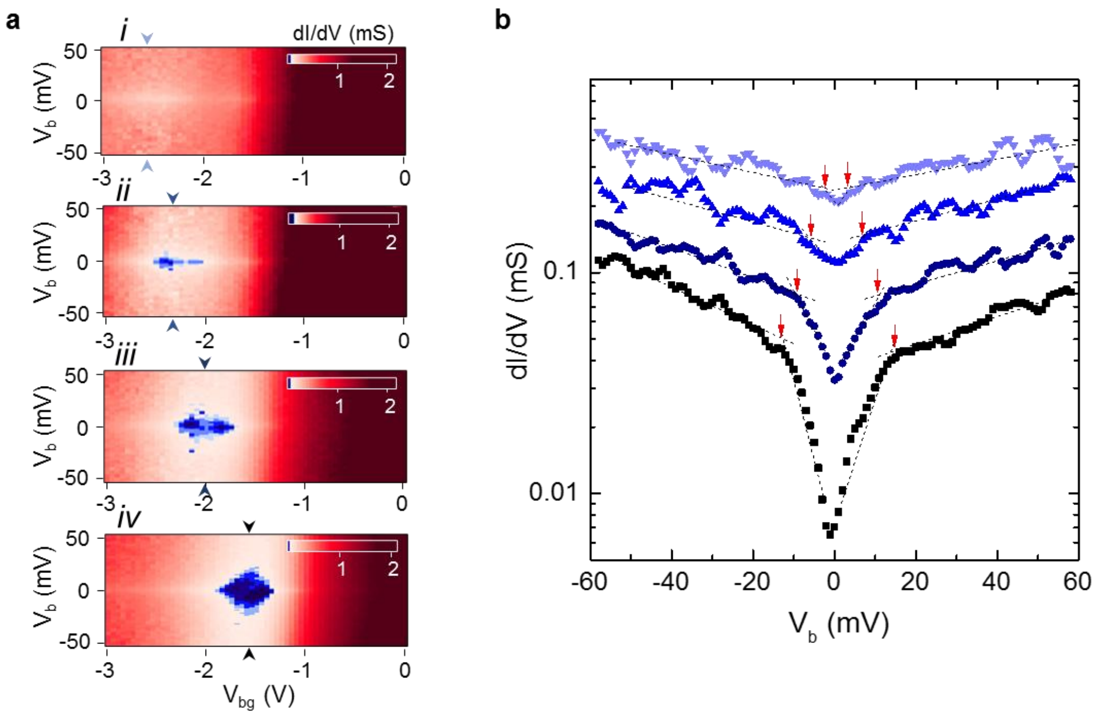

Supplementary Figure 17. Differential conductance in displacement field. **a** Color maps of dI/dV as functions of V_b and V_{bg} for the 9 nm hBSTS at different V_{tg} of (*i*) 1.5 V, (*ii*) 1 V, (*iii*) 0.5 V, and (*iv*) 0 V. **b** dI/dV line profiles at charge neutrality point taken from the respective color maps, as indicated by the blue arrows in **a**. Red arrows in **b** point to the turn-on voltages at the respective D.

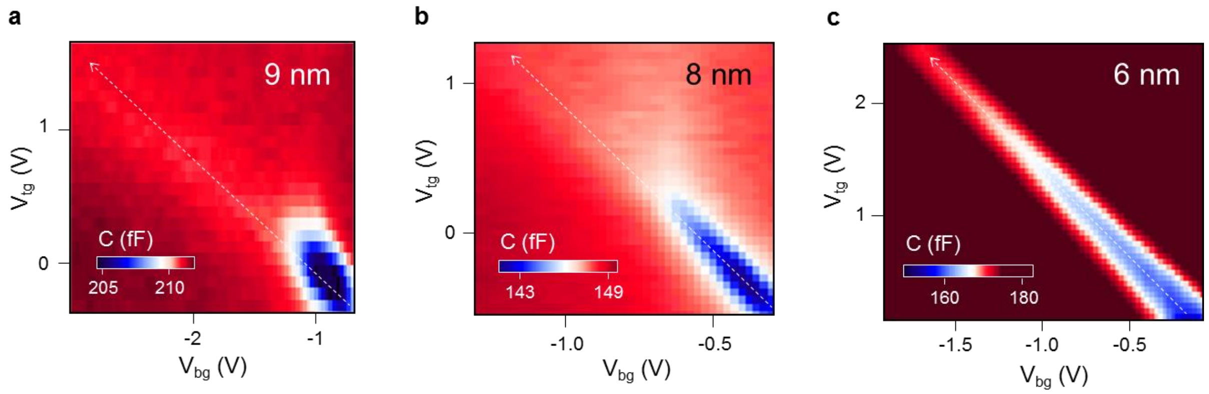

Supplementary Figure 18. Thermodynamic density of states in displacement field. Color maps of total capacitance (C) as functions of dual-gate voltages for the **a** 9, **b** 8, and **c** 6 nm hBSTS. The dashed line arrows in the figures point to the direction of the D applied at zero charge density.

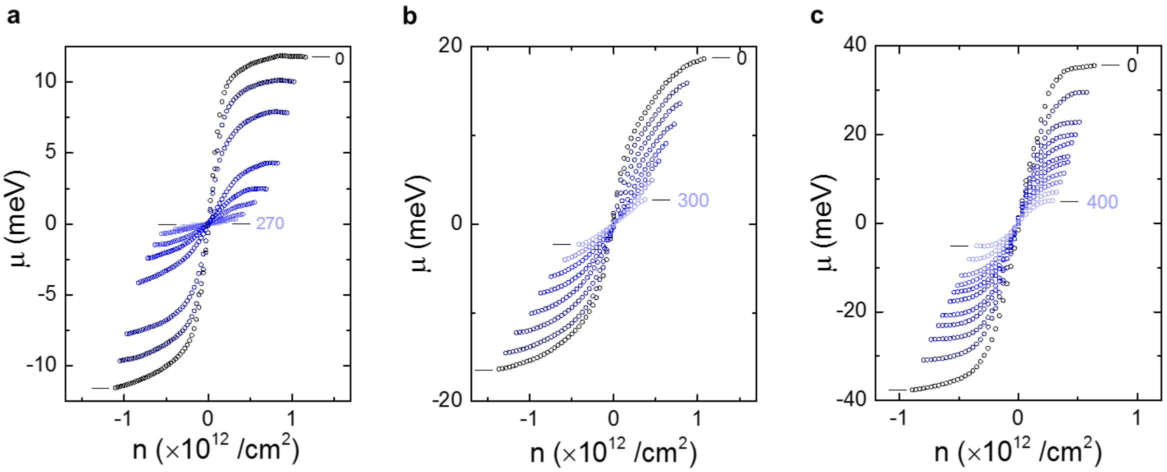

Supplementary Figure 19. Chemical potentials of the surface hybridization gap in displacement field. Plots of μ(n) *versus* n calculated from C_Q at different D for the **a** 9, **b** 8, and **c** 6 nm hBSTS. The surface hybridization gaps at different D were determined from the step height of the μ(n) plots.

VII Potential Topological Materials for Topological Transistors

Realization of electric field mediated topological quantum phase transitions offers great potential towards practical applications of topological quantum devices, such as topological transistors [18]. Switching topological phases for controllable on/off edge channels by electric field is desirable due to its high reversibility as compared to doping modulations, and lattice distortions by applying strain or pressure. As the proposed topological transistors rely on electric field-effect, a few criteria need to be satisfied. Firstly, the critical field E_c should not exceed the breakdown field for dielectrics, and more sufficiently smaller E_c for durability. Secondly, the difference of conductance response in trivial and topological states should be large enough to distinguish both states, which means the large on/off ratio. For the topological transistor, the "on" state conductance is fixed to the quantized value at $e^2/2h$. Therefore, the "off" insulating state determines the on/off ratio. Another important parameter is the size of this bulk surface gap, which determines the temperature limit of the topological edge states. From the electric field response of our hBSTS, we demonstrated a relatively small displacement field of ~0.25-0.8 V/nm with a surface hybridization gap of ~20-80 meV for 9-6 nm hBSTS. Also, the more than one order of magnitude change in ρ_{xx} between "on" and "off" states is highly promising for the proposed application. Supplementary Table 4 summarizes the theoretical and experimental parameters for electric field mediated topological phase transition in different types of topological systems reported in the literature [18-28].

Supplementary Table 4. Comparison of electric field mediated topological phase transition for different topological materials. The theoretical/experimental parameters, such as bulk surface gap (Δ), disorder strength (W), and gap-closing field (E_c) are included in the table.

**Materials**	**Topology**	**Δ (meV)**	**W (meV)**	**E_C (V/nm)**	**Refs**
HgTe/CdTe	2D TI	~40	~50	>0.003*	[19,20]
InAs/GaSb	2D TI	3-9	<10	~0.1	[21]
Strained InAs/InGaSb	2D TI	~20	-	-	[22]
Na_3Bi	2D TI	~300	-	1.12	[23]
$ZrTe_5$	Dirac semimetal	10-80	-	~0.07	[24]
Bi(111)	2D TI	~320	-	21*	[25]
Graphene/WSe_2	2D TI	~0.25	-	0.02	[26]
Monolayer 1T'-TMDs	Semimetal				
MoS_2		~80	-	1.42*	[18]
WTe_2	2D TI	45-55	-	-	[27,28]
Phosphorene	semiconductor	50-300	-	~1.1-3.0*	[29]
hBSTS	3D TI	~16-80	<5	~0.25-0.8	This work

*Based on theoretical calculations

Supplementary References

1. Frantzeskakis, E. et al. Dirac states with knobs on: Interplay of external parameters and the surface electronic properties of three-dimensional topological insulators. *Phys. Rev B* **91**, 2045134 (2015).
2. Arakane, T. et al. Tunable Dirac cone in the topological insulator $Bi_{2-x}Sb_xTe_{3-y}Se_y$. *Nat. Comm.* **3**, 636 (2012).
3. Liu, C. X. et al. Oscillatory crossover from two-dimensional to three-dimensional topological insulators. *Phys. Rev B* **81**, 041307(R) (2010).
4. Cao, G. et al. Rhombohedral Sb_2Se_3 as an intrinsic topological insulator due to strong van der Waals interlayer coupling. *Phys. Rev B* **97**, 075147 (2018).
5. Li, Z. et al. High thermoelectric performance of few-quintuple Sb_2Te_3 nanofilms. *Nano Energy* **43**, 285-290 (2018).
6. Roth, A., Brune, C., Buhmann, H., Molenkamp, L. W., Maciejko, J., Qi, X.-L. & Zhang, S.-C. Nonlocal transport in the quantum spin Hall state. *Science* **325**, 294-297 (2009).
7. Yang, Z. & Han, J. H. Landau level states on a topological insulator thin film. *Phys. Rev B* **83**, 045415 (2011).
8. Zyuzin, A. A. & Burkov, A. A. Thin topological insulator film in a perpendicular magnetic field. *Phys. Rev B* **83**, 195413 (2011).
9. Pertsova, A., Canali, C. M. & MacDonald, A. H. Thin films of a three-dimensional topological insulator in a strong magnetic field: microscopic study. *Phys. Rev. B* **91**, 075430 (2015).
10. Chong, S. K., Tsuchikawa, R., Harmer, J., Sparks, T. D. & Deshpande, V. V. Landau levels of topologically-protected surface states probed by dual-gated quantum capacitance. *ACS Nano* **14**, 1158-1165 (2020).

11. Zyuzin, A. A., Hook, M. D. & Burkov, A. A. Parallel magnetic field driven quantum phase transition in a thin topological insulator film. *Phys. Rev. B* **83**, 245428 (2011).
12. Zhou, B. Lu, H.-Z., Chu, R.-L., Shen, S.-Q. & Niu, Q. Finite size effects on helical edge states in a quantum spin-Hall system. *Phys. Rev. Lett.* **101**, 246807 (2008).
13. Liu, G., Zhou, G. & Chen, Y.-H. Modulation of external electric field on surface states of topological insulator Bi_2Se_3 thin films. *Appl. Phys. Lett.* **101**, 223109 (2012).
14. Kim, M., Kim, C. H., Kim, H.-S. & Ihm, J. Topological quantum phase transitions driven by external electric fields in Sb_2Te_3 thin films. *Proc. Natl. Acad. Sci. U.S.A.* **109**, 671-674 (2012).
15. Wang, J., Lian, B. & Zhang, S.-C. Electrically tunable magnetism in magnetic topological insulators. *Phys. Rev. Lett.* **115**, 036805 (2015).
16. Chong, S. K., Han, K. B., Sparks, T. D. & Deshpande, V. V. Tunable coupling between surface states of a three-dimensional topological insulator in the quantum Hall regime. *Phys. Rev. Lett.* **123**, 036804 (2019).
17. Breunig, O., Wang, Z., Taskin, A. A., Lux, J., Rosch, A. & Ando, Y. Gigantic negative magnetoresistance in the bulk of a disordered topological insulator. *Nat. Comm.* **8**, 15545 (2017).
18. Qian, X., Liu, J., Fu, L., Li, J. Quantum spin Hall effect in two-dimensional transition metal dichalcogenides. *Science* **346**, 1344-1347 (2014).
19. Yang, W., Chang, K. & Zhang, S.-C. Intrinsic spin Hall effect induced by quantum phase transition in HgCdTe quantum wells. *Phys. Rev. Lett.* **100**, 056602 (2008).
20. Maciejko, J., Qi, X.-L. & Zhang, S.-C. Magnetoconductance of the quantum spin Hall state. *Phys. Rev. B* **82**, 155310 (2010).

21. Qu, F. et al. Electric and magnetic tuning between the trivial and topological phases in InAs/GaSb double quantum wells. *Phys. Rev. Lett.* **115**, 036803 (2015).

22. Du, L., Li, T., Lou, W., Wu, X., Liu, X., Han, Z., Zhang, C., Sullivan, G., Ikhlassi, A., Chang, K., Du, R. Tuning edge states in strained-layer InAs/GaInSb quantum spin Hall insulators. *Phys. Rev. Lett.* **119**, 056803 (2017).

23. Collins, J. L. et al. Electric-field-tuned topological phase transition in ultrathin Na_3Bi. *Nature* **564**, 390-394 (2018).

24. Vaswani, C. et al. Light-driven Raman coherence as a nonthermal route to ultrafast topology switching in a Dirac semimetal. *Phys. Rev. X* **10**, 021013 (2020).

25. Sawahata, H., Yamaguchi, N., Kotaka, H. & Ishii, F. Electric field dependence of topological edge states in one-bilayer Bi(111): A first-principles study. *e-J. Surf. Sci. Nanotechnol.* **16**, 427-430 (2018).

26. Island, J. O. et al. Spin-orbit-driven band inversion in bilayer graphene by the van der Waals proximity effect. *Nature* **571**, 85-89 (2019).

27. Tang, S. et al. Quantum spin Hall state in monolayer 1T'-WTe_2. *Nat. Phys.* **13**, 683-687 (2017).

28. Wu, S., Fatemi, V., Gibson, Q. D., Watanabe, K., Taniguchi, T., Cava, R. J., Jarillo-Herrero, P. Observation of the quantum spin Hall effect up to 100 kelvin in a monolayer crystal. *Science* **359**, 76-79 (2018).

29. Liu, Q., Zhang, X., Abdalla, L. B., Fazzio, A., Zunger, A. Switching a normal insulator into a topological insulator *via* electric field with application to phosphorene. *Nano Lett.* **15**, 1222-1228 (2015).
